# A181 GEOGRAPHIC IMPACT ON SCREENING AND DEVELOPMENT OF CERVICAL NEOPLASIA IN INFLAMMATORY BOWEL DISEASE

**DOI:** 10.1093/jcag/gwae059.181

**Published:** 2025-02-10

**Authors:** Q Goddard, S Coward, C Seow, S Bertazzon, G G Kaplan

**Affiliations:** University of Calgary, Calgary, AB, Canada; University of Calgary, Calgary, AB, Canada; University of Calgary, Calgary, AB, Canada; University of Calgary, Calgary, AB, Canada; University of Calgary, Calgary, AB, Canada

## Abstract

**Background:**

While the risk of cancer in inflammatory bowel disease (IBD) is elevated, studies indicate a lower odds of gynecological cancers. Cervical cancer has become relatively preventable as a result of screening programs. However, geographic access impacts screening, possibly resulting in higher rates of cancer in rural areas.

**Aims:**

To investigate in the IBD population: (1) the odds of cervical neoplasia (squamous intraepithelial neoplasia grade III or cervical cancer) compared to matched controls, (2) screening rates (Pap smears), and (3) impact of urban vs rural residence on these estimates.

**Methods:**

We conducted a population-based matched cohort study using administrative healthcare databases in Alberta to identify females with IBD (*n*=22,245), age- and sex-matched 10-to-1 to controls (*n*=161,070) from fiscal years 2003–2021. The Alberta Cancer Registry provided morphology and diagnosis date for cervical neoplasia. Physician Claims provided Pap smears. Screening rates were defined as Pap smears per person-year (PY), with eligible Pap smears being those received by individuals aged 21–69, as per provincial screening guidelines. The Provincial Registry provided annual geographic data indicating urban vs rural residency. Average annual percentage change (AAPC) in screening and incidence of cervical neoplasia was calculated using Poisson regression. Conditional logistic regression compared cervical neoplasia in cases and controls, reported as odds ratios (ORs) and 95% confidence intervals (CIs), and evaluated rurality as an effect modifier using an interaction term. Two-sample t-tests compared mean Pap smears per PY between urban and rural populations.

**Results:**

Females with IBD have lower odds of both cervical cancer (OR: 0.65; 95%CI: 0.47, 0.92) and neoplasia (OR: 0.76; 95%CI: 0.68, 0.84) compared to controls, but rural status was not a modifier (*p*=0.38). Screening rates in those with IBD were not significantly different from controls (*p=*0.49). Rural individuals with IBD were screened less than their urban counterparts (0.086 vs 0.30 Pap smears per PY, *p*<0.001), and a similar pattern was observed in controls (0.087 vs 0.29 Pap smears per PY, *p*<0.001). The proportion of eligible individuals with IBD receiving Pap smears decreased over time (AAPC: −5.15; 95%CI: −5.30, −4.99), while the diagnosis of cervical neoplasia was stable (AAPC: −0.14; 95%CI: −2.03, 1.79).

**Conclusions:**

Individuals with IBD had lower odds of cervical neoplasia and cancer, regardless of rural vs urban status. Screening was lower for rural individuals in both IBD and non-IBD populations. Although screening rates declined in individuals with IBD, the detection of cervical neoplasia remained stable. Future studies should explore barriers to and timing of screening, especially in rural areas, as well as the reasons for the reduced risk of cervical neoplasia in IBD.

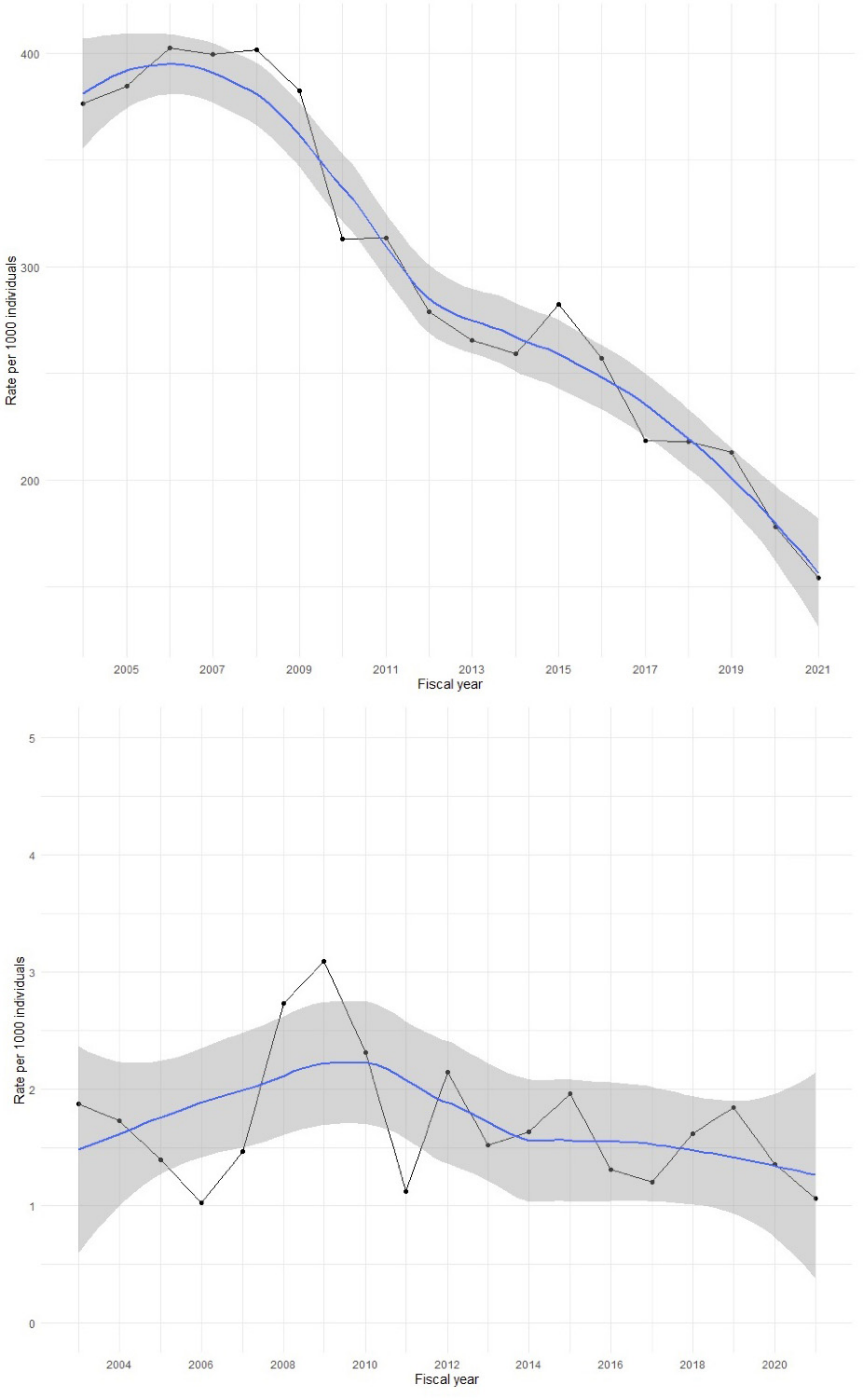

Yearly proportion of eligible individuals receiving a Pap smear in the IBD population (top) and yearly rate of cervical neoplasia diagnoses (bottom) in individuals with IBD.

**Funding Agencies:**

CIHR

